# The Relativistic Boltzmann Equation and Two Times

**DOI:** 10.3390/e22080804

**Published:** 2020-07-22

**Authors:** L. P. Horwitz

**Affiliations:** 1 School of Physics, Tel Aviv University, Ramat Aviv 69978, Israel; larry@tauex.tau.ac.il; 2 Department of Physics, Bar Ilan University, Ramat Gan 52900, Israel; 3 Department of Physics, Ariel University, Ariel 40700, Israel

**Keywords:** SHP theory, invariant world time, Einstein-Maxwell time, relativistic Boltzmann equation, antiparticles, entropy flow

## Abstract

We discuss a covariant relativistic Boltzmann equation which describes the evolution of a system of particles in spacetime evolving with a universal invariant parameter τ. The observed time *t* of Einstein and Maxwell, in the presence of interaction, is not necessarily a monotonic function of τ. If t(τ) increases with τ, the worldline may be associated with a normal particle, but if it is decreasing in τ, it is observed in the laboratory as an antiparticle. This paper discusses the implications for entropy evolution in this relativistic framework. It is shown that if an ensemble of particles and antiparticles, converge in a region of pair annihilation, the entropy of the antiparticle beam may decreaase in time.

## 1. Covariant Mechanics

Stueckelberg, in 1941 [1], wrote that the worldline of a particle in spacetime can be thought of as generated by an *event*, a point in spacetime, moving according to dynamical laws and generating such a worldline. In this way, one can write a dynamical Hamiltonian evolution for the event and achieve a covariant form for classical mechanics, where the eight dimensional phase space consists of $\left\{x^{\mu }\right\}$ and $\left\{p_{\mu }\right\}$, for μ=0,1,2,3. For a free particle, he proposed a Lorentz invariant Hamiltonian of the form

(1) $$K=\eta^{\mu \nu }\frac{p_{\mu }p_{\nu }}{2M}$$

where the metric $\eta^{\mu \nu }=\mathrm{diag}\left(-1,+1,+1,+1\right)$ may raise indices (we take units for which the velocity of light c=1), and its inverse, $\eta_{\mu \nu }$ can lower indices. The Hamilton equations, applied to this Hamiltonian results in

(2) $${\dot{x}}^{\mu }=\frac{\partial K}{\partial p_{\mu }}=\eta^{\mu \nu }\frac{p_{\nu }}{M}=\frac{p^{\mu }}{M},$$

where the dot indicates differentiation with respect to the invariant parameter τ and, for the free particle,

(3) $${\dot{p}}_{\mu }=-\frac{\partial K}{\partial x^{\mu }}=0.$$

From (2), we see that

(4) $${\dot{x}}^{\mu }{\dot{x}}_{\mu }=\frac{dx^{\mu }dx_{\mu }}{{\left(d\tau \right)}^{2}}=\frac{p^{\mu }p_{\mu }}{M^{2}}.$$

By the usual definition, we take

(5) $$p^{\mu }p_{\mu }=-E^{2}+p^{2}=-m^{2},$$

and, since the proper time squared is defined as

(6) $$ds^{2}=dt^{2}-dx^{2},$$

with $dx^{\mu }dx_{\mu }=-dt^{2}+dx^{2}$, (4) implies that

(7) $$\frac{ds^{2}}{d\tau^{2}}=\frac{m^{2}}{M^{2}}.$$

Since *E* and p are independent variables, the quantity *m*, the mass of the particle is necessarily a dynamical variable, and the theory is therefore called *off-shell* (the “shell” corresponds to the surface $E^{2}-p^{2}$, usually taken to be a constant, the given particle mass squared). We see from (4) that the particle proper time and the invariant parameter τ coincide when $m^{2}=M^{2}$, called *on shell*. We further remark that the Hamilton Equation (2) implies that the proper time (6) is essentially dynamical, since *t* and *x* obey equations of motion, and therefore, in this framework, it is not a suitable parameter to describe the motion. The theory therefore involves *two times*, one the invariant universal time, essentially the time of Newton [2] and the other the *observed* time *t* of Einstein and Maxwell.

In Stueckelberg’s original paper [1], he envisaged the possibility of the world line of a particle as starting as a free particle, straight and increasing in *t*, but then, under interaction, curving continuously to return in the negative direction of *t*. From this he observed, as remarked above, that *t* is not single valued, and consequently the introduction of a new parameter along the motion is necessary.

This configuration can be understood as *pair annihilation*; it was already known at that time [3] that a particle running backwards in time can be understood, and observed, as an antiparticle going forward in time. This phenomenon occurs in the solutions of the Dirac equation [3], where the wave function of a particle going backward in time, under charge conjugation, describes an antiparticle moving forward in time. Dirac [3], in this way, discovered the positron, the antiparticle of the electron.

Stueckelberg therefore called this configuration *pair annihilation in classical mechanics.*

The conclusions we have described above are also valid for guage invariant electromagnetic interaction for which the Hamiltonian is

(8) $$K=\eta^{\mu \nu }\frac{\left(p_{\mu }-eA_{\mu }\right)\left(p_{\nu }-eA_{\nu }\right)}{2M},$$

if $A_{\mu }$ is assumed independent of τ. In the same way as above, one finds that [1]

(9) $$\frac{ds^{2}}{d\tau^{2}}=\frac{\left(p_{\mu }-eA_{\mu }\right)\left(p^{\mu }-eA^{\mu }\right)}{2M},$$

which (as in (7) without further interaction or explicit τ dependence in the gauge field) cannot generate classical pair annihilation. Thus, the usual 4D electromagnetism does not generate classical pair annihilation. However, just as in the standard Schrödinger equation, where the $A_{0}$ component of the electromagnetic potential arises from the time derivative of the wave function, an additional potential is generated in the gauge transformation of the wave function of the Stueckelberg-Schrödinger equation [1] corresponding to the quantum theory associated with the Hamiltonian (8), leading to a 5D electrodynamics [4,5] (with, in general, τ dependent fields). In the classical limit, such a theory can achieve pair annihilation. We shall not discuss this subject further here, but turn to the many body problem.

In 1973, Horwitz and Piron [6] generalized the Stueckelberg theory to be applicable to many body systems by postulating that the parameter τ is universal, as for Newtonian time [2]. They were then able to solve the classical relativistic two-body central potential problem with a Hamiltonian of the form

(10) $$K=\frac{{p_{1}}_{\mu }{p_{1}}_{\mu }}{2M_{1}}+\frac{{p_{2}}^{\mu }{p_{2}}^{\mu }}{2M_{2}}+V\left(x_{1}-x_{2}\right),$$

where the potential term is a function of the invariant ${\left(x_{1}-x_{2}\right)}^{\mu }{\left(x_{1}-x_{2}\right)}_{\mu }$, in some generality. The corresponding quantum two body relativistic central potential problem was solved later by Arshansky and Horwitz [7].

One may think of the many event system as a many particle system since each event traces out a world line that can be identified with a particle [8,9]. In this sense we can formulate the notion of probability distributions and, eventually, a Boltzmann equation [10].

## 2. Boltzmann Equation

As we have mentioned, an event, in the dynamical sense under consideration, in the Minkowski space, with the properties of four-momentum as well as spacetime position, associated with the parameter *M*, can, through its trajectory (world line), be associated with a particle. In the same way, many events can be associated with many particles according to the world lines generated [8]. Orbits in the phase space cannot cross since the motion satisfies first order equations. By the postulate of Horwitz and Piron [6] ineractions between these particles are correlated by the universal invariant world time τ (discussions with E.C.G. Sudarshan [11]). The two particle interaction Hamiltonian (10) can be generalized to the N body system in the form

(11) $$K=\Sigma_{i}\frac{{p_{i}}_{\mu }{p_{i}}^{\mu }}{2M_{i}}+V\left(x_{1},x_{2}\cdots x_{N}\right),$$

with *V* a scalar function (its dependence on *x* may be such, as in (10), that it is invariant). For the quantum theory, the wave function $\psi_{\tau }\left(x_{1},x_{2}\cdots x_{N}\right)$ satisfies the Stueckelberg-Schrödinger equation [1]

(12) $$\frac{\partial \psi_{\tau }}{d\tau }=K\psi_{\tau },$$

with *K* given by (11). The wave functions have the same local interpretation as for the non-relativistic case, i.e.,

(13) $$p\left(x_{1},x_{2},\cdots x_{N}\right)={\left|\psi_{\tau }\left(x_{1},x_{2},\cdots x_{N}\right)\right|}^{2}$$

is the probability density per unit (*N* dimensional) volume $d^{4}x_{1}d^{4}x_{2}\cdots d^{4}x_{N}$ to find the events in the configuration $x_{1},x_{2},\cdots x_{N}$. We remark that in this framework, the momentum operator, unlike the more complicated form of the Newton-Wigner operator (*) Derived by taking into account the measure $\frac{d^{3}p}{2E}$ in the momentum space scalar product for the Klein-Gordon on mass-shell theory. [10,12], is represented on the configuration space as

(14) $${{x^{\mu }}_{i}}^{op}=-i\frac{\partial }{\partial {p_{i}}_{\mu }}.$$

For an arbitrary operator *A* on the Hilbert space $L^{2}\left(R^{4};d^{4}x\right)$ of wavefunctions $\left\{\psi_{\tau }\left(x_{1},x_{2}\cdots x_{N}\right)\right\}$, we may assume with Weyl [13,14] that the operator valued set $e^{i\Sigma_{i}{k_{i}}_{\mu }{x_{i}}^{\mu }+{j_{i}}^{\mu }{p_{i}}_{\mu }}$ is complete, so that we can write

(15) $$A=\int d^{4}k_{1}d^{4}k_{2}\cdots d^{4}k_{N}d_{1}^{j},d^{4}j_{2}\cdots d^{4}j_{N}A\left(k_{1},k_{2}\cdots k_{N},j_{1},j_{2}\cdots j_{N}\right)e^{i\Sigma_{i}{k_{i}}_{\mu }{x_{i}}^{\mu }+{j_{i}}^{\mu }{p_{i}}_{\mu }},$$

where the canonical operators satisfy (we take ℏ=1)

(16) $$\left[{x_{i}}^{\mu },{p_{j}}_{\nu }\right]=i\delta_{ij}{\delta^{\mu }}_{\nu },$$

and the coefficients $A(k_{1},k_{2}\cdots k_{N},j_{1},j_{2}\cdots j_{N})$ are the numerical valued representers of the operator *A*.

In a state described by the density matrix ρ, for which the expectation value of *A* is given by

(17) $$<A{>}_{\rho }=Tr\left(\rho A\right),$$

it follows that

(18) $$\begin{array}{cc} <A> & =\int d^{4}k_{1}d^{4}k_{2}\cdots d^{4}k_{N}d^{4}j_{1},d^{4}j_{2}\cdots d^{4}j_{N}\\ \; & A\left(k_{1},k_{2}\cdots k_{N},j_{1},j_{2}\cdots j_{N}\right)Tr\left(\rho e^{i\Sigma_{i}{k_{i}}_{\mu }{x_{i}}^{\mu }+{j_{i}}^{\mu }{p_{i}}_{\mu }}\right). \end{array}$$

We now wish to discuss one-particle, two-particle.... states of identical particles corresponding to the type of measurements to be performed. To do this, it is convenient to describe the many-body system in term of annihilation-creation operators [14,15], so that a general operator can be represented as a sum of *s*-body constituents as

(19) $$A=\Sigma_{s=1}^{N}\frac{1}{s!}\int d^{4}x_{d}^{4}x_{2}\cdots d^{4}x_{s}\psi^{†}\left(x_{1}\right)\psi^{†}\left(x_{2}\right)\cdots \psi^{†}\left(x_{s}\right)A_{s}\psi \left(x_{1}\right)\psi \left(x_{2}\right)\cdots \psi \left(x_{s}\right),$$

where $A_{s}$ is an operator defined on the *s*-body subspace.

Consider first the one body case, s=1. Then, from (17),

(20) $$<A_{1}>=\int d^{4}x\int d^{4}kd^{4}jA_{1}\left(k,j\right)exp\left(ik\cdot \left(x+\frac{j}{2}\right)\right)Tr\left(\rho \psi^{†}\left(x+j\right)\right),$$

where the center dot signifies the Lorentz scalar product. As in the work of Dewdney et al. [16], we define the one-particle Wigner function [17]

(21) $$\begin{array}{cc} {f_{1}}^{W}\left(x,p\right) & =\frac{1}{{\left(2\pi \right)}^{4}}\int d^{4}je^{-ij\cdot p}Tr\left(\rho \psi^{†}\left(x-\frac{j}{2}\right)\right)\psi \left(x+\frac{j}{2}\right))\\ \; & =\frac{1}{{\left(2\pi \right)}^{4}}\int d^{4}ke^{-ik\cdot x}Tr\left(\rho \psi^{†}\left(p-\frac{k}{2}\right)\psi \left(p+\frac{k}{2}\right)\right). \end{array}$$

We can then write (20) as

(22) $$<A_{1}>=\int d^{4}xd^{4}pA_{1}\left(x,p\right){f_{1}}^{W}\left(x,p\right).$$

It is clear from (21) that the function ${f_{1}}^{W}\left(x,p\right)$ is not, in general positive, but nevetheless provides a state dependent measure for the expectation value of the one body operator $A_{1}$ in terms of the numerical valued kernel $A_{1}\left(x,p\right)$ on the phase space. It has the property, furthermore, that the (boundary) contraction

(23) $$\int d^{4}x{f_{1}}^{W}\left(x,p\right)=Tr(\rho \psi^{†}\left(p\right)\psi \left(p\right)\geq 0,$$

and its normalization is determined by

(24) $$\begin{array}{cc} \int d^{4}xd^{4}p{f_{1}}^{W}\left(x,p\right) & =\int d^{4}xTr\left(\rho \psi^{†}\left(x\right)\psi \left(x\right)\right)\\ \; & =NTr\rho =N. \end{array}$$

Let us define the Fourier transform of (21) from *x* to *k*,

(25) $${f_{1}}^{W}\left(k,p\right)=\int d^{4}xe^{-ik\cdot x}{f_{1}}^{W}\left(x,p\right)=Tr(\rho \psi^{†}\left(p-\frac{k}{2}\right)\psi \left(p+\frac{k}{2}\right).$$

The Wigner function provides a useful measure for describing the quantum state, very close to that of a classical measure on the phase space, and, in fact, satisfies an equation of motion, the Boltzmann equation, similar in form to that of the classical theory.

We now turn to study the dependence of this function on the invariant world time. The world time dependence is determined by that of the density matrix which, as for any observable, is

(26) $$\partial_{\tau }\rho =i\left[K,\rho \right].$$

Cycling under the trace, we have

(27) $$\partial_{\tau }{f_{1}}^{W}\left(k,p\right)=iTr\rho \left[K,\psi^{†}\left(p-\frac{k}{2}\right)\psi \left(p+\frac{k}{2}\right)\right].$$

We now assume that *K* has the form

(28) $$K=K_{0}+V,$$

where, as in Stueckelberg’s original work (but here in second quantized form for the many-body system [18])

(29) $$K_{0}=\frac{1}{2M}\int d^{4}x\psi^{†}\left(x\right)p_{\mu }p^{\mu }\psi \left(x\right)$$

and

(30) $$V=\frac{1}{2}\int d^{4}x^{\prime }d^{4}x^{\prime \prime }\psi^{†}\left(x^{\prime }\right)\psi^{†}\left(x^{\prime \prime }\right)V\left(x^{\prime }-x^{\prime \prime }\right)\psi \left(x^{\prime \prime }\right)\psi \left(x^{\prime }\right).$$

The commutator of $K_{0}$ with the two body potential does not change the number of annihilation-creation operators in *V*, and therefore adds a *two event* term to the time derivative of the Wigner function. One finds [10,14] that

(31) $$\partial {f_{1}}^{W}\left(k_{1},p_{1}\right)={L_{1}}^{0}{f_{1}}^{W}\left(k_{1},p_{1}\right)+\int d^{4}p_{2}d^{4}k_{2}\delta^{4}\left(k_{2}\right)L_{12}{f_{2}}^{W}\left(k_{1}p_{1},k_{2}p_{2}\right),$$

where

(32) $${L_{1}}^{0}=-\frac{i}{M}\left(k_{1}\cdot p_{1}\right)$$

and, for

(33) $$\tilde{V}\left(\ell \right)=\frac{1}{{\left(2\pi \right)}^{4}}\int d^{4}xe^{-ix\cdot \ell }V\left(x\right),$$

we have

(34) $$\begin{array}{cc} L_{12} & =-i\int d^{4}\ell \tilde{V}\left(\ell \right)[\exp\left\{-\frac{\ell^{\mu }}{2}\left(\frac{\partial }{\partial {p_{1}}^{\mu }}-\frac{\partial }{\partial {p_{2}}^{\mu }}\right)\right\}\\ \; & -\exp\left\{\frac{\ell^{\mu }}{2}\left(\frac{\partial }{\partial {p_{1}}^{\mu }}-\frac{\partial }{\partial {p_{2}}^{\mu }}\right)\right\}]\exp\left\{-\ell^{\mu }\left(\frac{\partial }{\partial {k_{1}}^{\mu }}-\frac{\partial }{\partial {k_{2}}^{\mu }}\right)\right\}. \end{array}$$

In (31),

(35) $$\begin{array}{cc} {f_{2}}^{W}\left(k_{1},p_{1},k_{2},p_{2}\right) & =\int d^{4}x_{1}d^{4}x_{2}e^{-ik_{1}\cdot x_{1}-ik_{2}\cdot x_{2}}{f_{2}}^{W}\left(x_{1}p_{1},x_{2}p_{2}\right)\\ \; & =Tr(\rho \psi^{†}\left(p_{1}-\frac{k_{1}}{2}\right)\psi^{†}\left(p_{2}-\frac{k_{2}}{2}\right)\\ \; & \times \psi \left(p_{2}+\frac{k_{2}}{2}\right)\psi \left(p_{1}+\frac{k_{1}}{2}\right)), \end{array}$$

where ${f_{2}}^{W}$ is defined as the *two-body Wigner function*.

The evolution of the one-body Wigner function therefore involves the two-body Wigner function. Continuing this process, each step applied to the *s*-body Wigner function involves the Wigner function for (s+1) events, building the so-called BBGKY hierarchy [10,19]. Since higher order correlations are expected to be small, the usual procedure is to replace the two event Wigner function by *collision* terms which induce transitions between events outside the one event distribution into the distribution and events in the one-event distribution to events which lie outside. The result is the Boltzmann equation, often a good approximation to the full evolution, here in relativistically covariant form [10].

We start by observing that the first term of (31) can be understood, in the original space (x,p), by noting that the classical Liouville theorem implies that (we write here f(x,p) for ${f_{1}}^{W}\left(x,p\right)$)

(36) $$f\left(x^{\mu }+\frac{p^{\mu }}{M}\delta \tau ,p^{\mu },\tau +\delta \tau \right)=f\left(x^{\mu },p^{\mu }\tau \right),$$

from which it follows immediately that

(37) $$\partial_{\tau }f\left(x,p\right)+\frac{p^{\mu }}{M}\frac{\partial }{x^{\mu }}f\left(x,p\right)=0$$

for the free flow of non-interacting events, in precise agreement with the first term of (31). Interactions would cause a departure from this flow, causing events to scatter out of or into the distribution f(x,p), conventionally called $D_{c}f\left(x,p\right)$, so that, both classically and quantum mechanically, we may write

(38) $$\partial_{\tau }f\left(x,p\right)+\frac{p^{\mu }}{M}\frac{\partial }{x^{\mu }}f\left(x,p\right)=D_{c}f\left(x,p\right).$$

Locally such processes correpond to momentum transfers due to collisions, i.e., for which, in two body scattering, an event at *x* with momentum $p^{\prime }$, scattering with another of momentum $p_{1}^{\prime }$ results in events with momenta $p_{1}$ and *p*, or an event with momentum *p* scatters with another of momentum $p_{1}$ to yield events with momentum $p_{1}^{\prime },p^{\prime }$. The first process, independently of $p_{1}^{\prime }$, brings an event into the distribution f(x,p), and the second, independently of $p_{1}$, removes and event from the distribution f(x,p). Multiplying the transition probability densities by the densities f(x,p) with appropriate momenta, one then finds for the scattering in and out processes,

(39) $${D_{c}}^{+}=\int d^{4}p_{1}d^{4}p_{1}^{\prime }d^{4}p^{\prime }\dot{P}\left(p_{1}^{\prime }p^{\prime }\to p_{1}p\right)f\left(x,p^{\prime }\right)f\left(x,p_{1}^{\prime }\right),$$

for events scattering into the distribution, and, for events scattering out,

(40) $${D_{c}}^{-}=\int d^{4}p_{1}d^{4}p_{1}^{\prime }d^{4}p^{\prime }\dot{P}\left(p_{1}p\to p_{1}^{\prime }p^{\prime }\right)f\left(x,p\right)f\left(x,p_{1}\right),$$

for the two types of contributions to $D_{c}f\left(x,p\right)$.

The scattering probalities for the two body problem with central potential can be written [20] in terms of total (conserved) four momentum $P=p_{1}+p_{2}$ and relative momentum $p_{r}=\frac{1}{2}\left(p_{1}-p_{2}\right)$, center of mass $\frac{1}{2}\left(x_{1}+x_{2}\right)$ and relative coordinate $x_{r}=x_{1}-x_{2}$, so that

(41) $$\dot{P}\left(Pp_{1}\to P^{\prime }p_{1}^{\prime }\right)=\delta^{4}\left(P-P^{\prime }\right)\dot{P}\left(p_{1}\to p_{1}^{\prime };P\right).$$

It then follow from (39) and (40) that

(42) $$\begin{array}{cc} {D_{c}}^{+} & =\int d^{4}p_{r}d^{4}p_{r}^{\prime }\dot{P}\left(p_{r}^{\prime }\to p_{r};P\right)f\left(x,p^{\prime }\right)f\left(x,p_{1}^{\prime }\right)\\ {D_{c}}^{-} & =\int d^{4}p_{r}d^{4}p_{r}^{\prime }\dot{P}\left(p_{r}\to p_{r}^{\prime };P\right)f\left(x,p\right)f\left(x,p_{1}\right). \end{array}$$

where

(43) $$\begin{array}{cc} P=2(p+p_{r})\;\; & \;\;p^{\prime }=p+p_{r}-p_{r}^{\prime }\\ p_{1}^{\prime }=p+p_{r}+p_{1}^{\prime }\;\; & \;\;p_{1}=2p_{r}+p. \end{array}$$

Using detailed balance, which follows from τ reversal and space-time reflection invariance,

(44) $$\dot{P}\left(p_{r}^{\prime }\to p_{r};P\right)=\dot{P}\left(p_{r}\to p_{r}^{\prime };P\right),$$

the relativistic Boltzman equation then becomes

(45) $$\begin{array}{cc} \partial_{\tau }f\left(x,p\right)+\frac{p^{\mu }}{M}\frac{\partial }{x^{\mu }}f\left(x,p\right) & =\int d^{4}p_{r}d^{4}p_{r}^{\prime }\dot{P}\left(p_{r}^{\prime }\to p_{1};P\right)\\ \; & \times \{f\left(x,p^{\prime }\right)f\left(x,p_{1}^{\prime }\right)-f\left(x,p\right)f\left(x,p_{1}\right)\}. \end{array}$$

The transition rate $\dot{P}$ is related to the differential cross section [20]. Assuming narrow distributions, one finds, in terms of experimentally observed cross sections that

(46) $$\begin{array}{cc} \partial_{\tau }f\left(x,p\right)+\frac{p^{\mu }}{M}\frac{\partial }{x^{\mu }}f\left(x,p\right) & =4\pi \int d^{3}p_{r}d^{3}p_{r}^{\prime }\frac{\left|p_{r}^{\prime }\right|}{M}\frac{d\sigma^{exp}}{d^{3}p_{1}}\left(p_{r}^{\prime }\to p_{r};P\right)\\ \; & \times \{f\left(x,p^{\prime }\right)f\left(x,p_{1}^{\prime }\right)-f\left(x,p\right)f\left(x,p_{1}\right)\}. \end{array}$$

The integrations on $d^{3}p$ are what remain after carrying out the $dp^{0}$ integrations on a wave packet. The relativistic cross section has dimension $TL^{2}$ (orthogonal to the axis of the beam), and $p^{0}$ dimension $T^{-1}$, leaving the cross section $\sigma^{exp}$ with the usual dimenion $L^{2}$.

## 3. Entropy Flow

We are now in a position to discuss the properties of entropy flow associated with the relativistic Boltzmann Equation (46). This result, that there are intrinsically two types of time, the *observable* time *t* associated with the Maxwell equations and Einstein’s formulation of special relativity (appearing in the Lorentz transformation), and the underlying universal parameter τ of dynamical evolution, as originally conceived by Newton [2], is a consequence of the Stueckelberg-Horwitz-Piron formulation of relativistic mechanics. * J.R. Fanchi [21] has discussed another approach to this theory based on the covariant current. We shall show that the relativistic Boltzmann equation implies a monotonic increase of entropy in the universal dynamical time τ. However, as pointed out in the original work of Stueckelberg [1], an event moving dynamically in the Minkowski spacetime may move in the negative direction of *t*; this phenomenon was identified by Stueckelberg as describing an antiparticle moving forward in *t*, the usual perception (as in the Maxwell equations) of time as the outcome of observation. Thus, for a beam of antiparticles, the entropy may appear to *decrease*.

Let us define the functional (following Boltzmann’s terminology [22])

(47) $$H=\int d^{4}xd^{4}pf\left(x,p,\tau \right)\ln f\left(x,p,\tau \right)=-S\left(\tau \right)/k_{B},$$

where S(τ) is the entropy and $k_{B}$ is Boltzmann’s constant. Then the derivative of *H*, using(46) and integration by parts of the spacetime derivatives,

(48) $$\begin{array}{cc} \frac{dH}{d\tau } & =\int d^{4}xd^{4}p\left[\ln f\left(x,p\right)+1\right]\int d^{4}p_{\tau }d^{4}p_{\tau }^{\prime }\dot{P}\left(p_{\tau }\to p_{\tau }^{\prime };P\right)\\ \; & \times \{f\left(x,p^{\prime }\right)f\left(x,p_{1}^{\prime }\right)-f\left(x,p\right)f\left(x,p_{1}\right)\}. \end{array}$$

Now, replace $d^{4}p_{\tau }$ by $\frac{d^{4}p_{1}}{16}$ and $p_{\tau }$ by $\frac{p_{1}-p}{2}$. The total energy momentum *P* entering into the binary collision process is invariant under interchange of *p*, $p_{1}$ and $p^{\prime }$, $p_{1}^{\prime }$. Interchanging these variables in the integrand and using the symmetry of $\dot{P}$ under time reversal and space inversion in relative coordinates, one obtains the same relation but with lnf(x,p) replaced by $\ln f(x,p_{1})$. Averaging these two forms and interchanging primed and unprimed momenta, with the detailed balance relation, we obtain

(49) $$\begin{array}{cc} \frac{dH}{d\tau } & =\frac{1}{64}\int d^{4}xd^{4}pd^{4}p_{1}d^{4}p_{1}^{\prime }\left[\ln f\left(x,p_{1}\right)f\left(x,p\right)-\ln f\left(x,p_{1}^{\prime }\right)f\left(x,p^{\prime }\right)\right]\\ \; & \times \left\{f\left(x,p^{\prime }\right)f\left(x,p_{1}^{\prime }\right)-f\left(x,p\right)f\left(x,p_{1}\right)\right\}\dot{P}\left(\left(\frac{p_{1}-p}{2}\to \frac{p_{1}^{\prime }-p^{\prime }}{2}\right);P\right). \end{array}$$

Since $\dot{P}\left(p_{\tau }\to p_{\tau }^{\prime };P\right)\geq 0$, and the remaining factor in the integrand is non-positive, we obtain

(50) $$\frac{dH}{d\tau }\leq 0.$$

We see that the relativistic Boltzmann equation implies that the entropy $S\left(\tau \right)=-k_{B}H\left(\tau \right)$ is non-decreasing for the flow of particles with binary collision interactions. According, however, to Stueckelberg’s original formulation, as we have discussed above, a free particle world line for which the generating event moves in the positive direction of *t*, as mentioned above, may turn and continue to move in the reverse direction of *t* due to interaction. This motion in the negative direction of *t* is interpreted, consistently with Dirac’s interpretation [3], as an *antiparticle* moving forward in *t*. The transition from forward to backward motion in *t*, in Stueckelberg’s view, can therefore be considered as a particle-antiparticle annihilation process. In the case of the flow of an ensemble of particles, such as a beam, if the particles encounter a region of interaction which can turn the flow back in time, and the entropy continues to increase in τ for the backward flowing beam, constituting a beam of antiparticles, one would observe the antiparticLe beam as *decreasing* in entropy, becoming less disordered as the beam approaches the annihilation region. This phenomenon may play a role in general relativity [23,24].

## 4. Conclusions

We have reviewed and discussed the derivation of the relativistic Boltzmann equation. The flow of an ensemble of particles governed by this equation is shown to obey the usual *H*-theorem implying a non-decreasing entropy in the usual definition of the time *t* (according to Maxwell and Einstein), as well as in the invariant world time τ, provided that there are no antiparticles involved. If antiparticles occur as part of a pair annihilation process, one may observe that the entropy flow of the antiparticle part of the system may have decreasing entropy in *t*, and therefore, even on a classical level, the generally expected increase of entropy may not be universally observed.
